# Evaluation of Denoising Performance of ResNet Deep Learning Model for Ultrasound Images Corresponding to Two Frequency Parameters

**DOI:** 10.3390/bioengineering11070723

**Published:** 2024-07-16

**Authors:** Hyekyoung Kang, Chanrok Park, Hyungjin Yang

**Affiliations:** 1 Department of Radiological Science, College of Health & Medical, Shingu University, 377, Gwangmyeong-ro, Jungwon-gu, Seongnam-si 13174, Republic of Korea; sonojjang@shingu.ac.kr; 2 Department of Radiological Science, College of Health Science, Eulji University, 553, Sanseong-daero, Sujeong-gu, Seongnam-si 13135, Republic of Korea; tigeaglepcr@eulji.ac.kr; 3 Department of Physics, Korea University, Sejong 30019, Republic of Korea

**Keywords:** ultrasound, frequency, deep learning, residual network (ResNet), quantitative analysis

## Abstract

Ultrasound imaging is widely used for accurate diagnosis due to its noninvasive nature and the absence of radiation exposure, which is achieved by controlling the scan frequency. In addition, Gaussian and speckle noises degrade image quality. To address this issue, filtering techniques are typically used in the spatial domain. Recently, deep learning models have been increasingly applied in the field of medical imaging. In this study, we evaluated the effectiveness of a convolutional neural network-based residual network (ResNet) deep learning model for noise reduction when Gaussian and speckle noises were present. We compared the results with those obtained from conventional filtering techniques. A dataset of 500 images was prepared, and Gaussian and speckle noises were added to create noisy input images. The dataset was divided into training, validation, and test sets in an 8:1:1 ratio. The ResNet deep learning model, comprising 16 residual blocks, was trained using optimized hyperparameters, including the learning rate, optimization function, and loss function. For quantitative analysis, we calculated the normalized noise power spectrum, peak signal-to-noise ratio, and root mean square error. Our findings showed that the ResNet deep learning model exhibited superior noise reduction performance to median, Wiener, and median-modified Wiener filter algorithms.

## 1. Introduction

Among the existing medical tools, such as computed tomography (CT), magnetic resonance imaging (MRI), positron emission tomography (PET), and ultrasonography, CT based on X-rays can be used to obtain depth information by rotating the X-ray tube using a contrast agent [1]. In this way, an accurate diagnosis can be obtained; however, the high radiation exposure and side effects of contrast agents may cause other problems and need to be resolved. MRI is also useful for acquiring soft-tissue information. Recently, a hybrid imaging system combining PET and MRI has been developed to acquire anatomical and functional information during a single examination [2]. MRI has many advantages but suffers from long scan durations, as well as loud scan sounds during signal acquisition and narrow bore size [3]. PET, which is another modality in nuclear medicine, is based on the acquisition of two gamma rays and can be used to obtain functional information from patients and for early detection of cancer lesions [4]. To generate two gamma rays, a radioisotope labeled with a radiopharmaceutical is injected into a patient. Thus, patients should consider radiation exposure, and the spatial resolution should be lower than that of CT and MRI images.

Ultrasound imaging is a noninvasive technique for lesion detection that involves various scan parameters. No radiation exposure and a low cost are some of its advantages, and it can be used to examine real-time internal vessel status, such as the carotid artery. In addition, ultrasound imaging involves collecting signals of various transmission refractions from the internal tissue using high-frequency sound waves. However, it suffers from noise, which degrades the image quality during the process of acquiring the transmitted signal. Noise reduction in ultrasound imaging is an important challenge. Generally, speckle and Gaussian noise are present in ultrasound imaging. Speckle noise is represented by a granular pattern and can degrade image quality, such as edge detection [5,6]. Gaussian noise is a statistical noise in digital imaging systems [7,8]. Many researchers have conducted studies on the denoising effect [9,10]. Lan et al. reduced the speckle noise in a real-time ultrasound image using a residual and mixed-attenuation mechanism network [9]. In addition, Mafi et al. proposed filtering algorithms in ultrasound imaging to remove noise fusion, such as speckle and Gaussian noise [10]. Conventional techniques for noise reduction broadly apply a filtering algorithm to spatial domains, such as median and Wiener filters, with a suitable mask size. Singh et al. implemented signal and similarity analysis between speckle noisy and ground truth images in ultrasound imaging and demonstrated the efficiency of spatial filtering algorithms in reducing the speckle noise distribution [11]. Kumar et al. evaluated the performance of median and Wiener filters in reducing Gaussian, salt-and-pepper, and speckle noises [12]. Furthermore, fusion filtering algorithms, such as the median modified Wiener filter (MMWF), which is converted to a median value based on the Wiener filter formula at the set mask size, are widely used to reduce the noise distribution. The MMWF is useful for preserving the edge signal and reducing the blurring effect when denoising degraded images. Park et al. suggested the MMWF algorithm for noise reduction in CT images [13]. In addition, Lee et al. proposed the MMWF algorithm to be applied to the acquired X-ray image, which was obtained under an experimental study that consisted of a CMOS-type X-ray system [14]. Computer-aided diagnosis (CAD) techniques based on deep convolutional neural networks (CNNs) have gradually become popular in the medical imaging field. Chang et al. reported that a two-stage CNN model exhibits a significant noise reduction effect and can perform simple image processing [15]. To detect accurate and various features based on deep learning algorithms, the layer depth of a network is a critical parameter. However, deeper network models are computationally expensive. In addition, prominent issues such as the vanishing and exploding gradients are liable to occur when performing backpropagation learning [16]. Thus, the learning performance would be degraded, and deep CNN is not an essential requirement. To overcome this limitation, He et al. developed a new alternative residual neural network (ResNet), which compensated for the limitations of deep CNN [17]. The ResNet model that uses residual blocks can reduce the training error rate and, consequently, achieve high performance. Thus, more features can be extracted as the depth of layers increases. Among the deep learning models for improving image quality in medical imaging, the ResNet model exhibits excellent performance in terms of the super-resolution imaging field [18].

The image resolution of ultrasound imaging depends on the frequency, which is one of the most important parameters for improving image quality. Foster et al. reported that a higher frequency can achieve better resolution but results in limited penetration depth owing to the attenuation and scattering of the ultrasound energy [19]. The sonographer must determine the appropriate frequency for the maximum depth to reach the target organ using ultrasound. For example, abdominal ultrasound examinations typically employ frequencies ranging from 2.5 to 3.5 MHz for deep and general imaging. In addition, a frequency of 5.0 MHz is used for vascular, breast, or pelvic imaging in the clinical ultrasound field. 

Despite substantial related research, studies on fusion noise reduction using a deep learning-based framework that addresses both low and high frequencies are limited. In this study, we generated images combined with Gaussian and speckle noises. To confirm the noise reduction effect, we devised an alternative algorithm based on the ResNet deep learning model. To validate the effectiveness of the deep learning algorithm in noise distribution reduction, conventional noise reduction algorithms, such as the median filter, Wiener filter, and MMWF algorithm (wherein the median and Wiener filters were fused), were used with quantitative analysis methods, such as the normalized noise power spectrum (NNPS), peak signal-to-noise ratio (PSNR), and root mean square error (RMSE). Thus, the aim of our study was to evaluate the noise reduction performance of a ResNet deep learning model at 3 and 5 MHz and make a comparison with conventional noise reduction filtering algorithms, such as the median filter, Wiener filter, and MMWF algorithm.

## 2. Materials and Methods

### 2.1. Dataset and ResNet Framework

**Figure 1:**
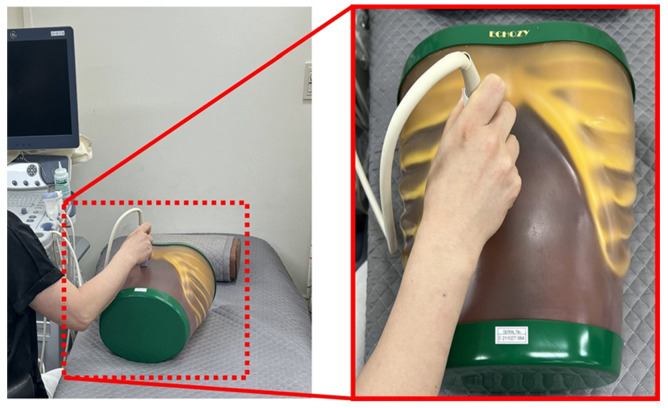
Experimental setup including acquisition of dataset at 3 and 5 MHz.

**Figure 2:**
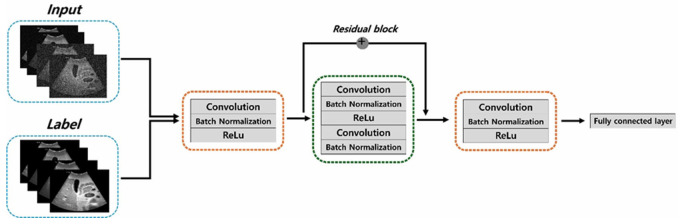
The ResNet model architecture with 16 residual blocks.

An Accuvix V10 instrument (Medison, Seoul, Korea) and an abdominal ultrasound phantom (US1, 41900-010, Ecozy, Kyoto, Japan) were used to obtain the ultrasound dataset (Figure 1). The total dataset comprised 1000 images acquired using the convex probe at 3 and 5 MHz. The scan conditions were 90% power, 95 dB dynamic range, 1 for edge enhancement, 0 factor for rejection, 2 numbers for the focus number, and 17 cm for the depth of field. Thus, the acquired images consisted of 500 slices at frequencies of 3 and 5 MHz each. To confirm the denoising effect, we added Gaussian and speckle noise with 0.01 and 0.1 standard deviations, respectively, to the ultrasound phantom images acquired at 3 and 5 MHz. Consequently, the dataset consisted of 500 slices of each noisy image with standard deviations of 0.01 and 0.1 at 3 MHz. Additionally, the images consisted of 500 slices of noisy images with standard deviations of 0.01 and 0.1 at 5 MHz. Furthermore, we applied conventional noise reduction filtering techniques, such as the median filter, Wiener filter, and MMWF algorithm, to the images; then, we compared the denoising performances of the conventional filter algorithms and the proposed method. Particularly, the MMWF algorithm proved to be efficient in reducing noise distribution in noisy images. Figure 2 shows a simple flowchart of the ResNet framework used in this study. The preprocessing steps included converting DICOM data to numpy format, resizing images, and normalizing data values from 0 to 1. The resulting dataset was then split into training, validation, and test sets in an 8:1:1 ratio, respectively. The ResNet network consisted of an encoder, a residual block, a decoder, and fully connected layer parts. The convolution calculation with a 3 × 3 kernel size, 1 for stride and 1 for padding, was implemented, and batch normalization and rectified linear unit (ReLU) were applied. The residual block layers consisting of 16 residual blocks in this study performed convolution, batch normalization, ReLu, convolution, and batch normalization. After the encoder and residual block process, the learning model was processed by the decoder and fully connected layer to generate the optimized noise reduction learning model in ultrasound imaging. The ResNet model has been designed to recognize images and detect objects; however, in this study, we used it for noise reduction. First, we input the noisy images, with 0.01 and 0.1 standard deviation values of Gaussian and speckle noise, respectively, into the proposed ResNet framework. Notably, a ResNet model could be applied with a residual block module that improved its learning accuracy and performance. In this study, the optimized network training model was obtained by the MSE loss function with an adaptive moment estimation (Adam) optimizer using Python 3.10.11 and PyTorch 1.12.1. Additionally, the learning rate was 1 × 10^−4^ with 300 epochs and a batch size of 2 using the AMD Ryzen 5 5600X CPU, 64 GB RAM, and NVIDIA GeForce RTX 4080 16 GB.

### 2.2. Quantitative Analysis

To validate the noise reduction efficiency of the deep learning model, we used quantitative analysis methods, such as NNPS, PSNR, and RMSE, under the set conditions. The NNPS, which contains information concerning the noise distribution in the acquired image, can be calculated as follows:

(1) $$NNPS=\frac{\frac{∆x∆y}{M\times N_{x}\times N_{y}}\sum_{m=0}^{M-1}{\left|\sum_{i=0}^{N_{x}-1}\sum_{j=0}^{N_{y}-1}\left(\left(I(x_{i},y_{i}\right)-\dot{I}(x_{i},y_{i})\right)\cdot e^{-2\pi i(ux_{i}+vy_{i})}\right|}^{2}}{{Average\;pixel\;value\;for\;mean\;ROI}^{2}},$$

where u and v are the spatial frequencies; $N_{x}$ and $N_{y}$ are the pixel numbers, and ∆x and ∆y are the pixel sizes along the x- and y- axes, respectively; $I(x_{i},y_{i})$ is the pixel value, and $\dot{I}(x_{i},y_{i})$ is the average pixel value. In the NNPS analysis, we acquired the average NNPS values in five different locations by drawing regions of interest to reduce the statistical error rate. Similarity analysis was performed using the PSNR and RMSE. The PSNR and RMSE were calculated as follows:

(2) $$PSNR=20\;\cdot \;{log}_{10}\left(\frac{{max}_{s}}{\sqrt{MSE}}\right),$$

(3) $$RMSE=\sqrt{MSE}=\sqrt{\sum_{i=1}^{m}\sum_{j=1}^{n}\frac{{\left({\hat{y}}_{i,j}-y_{i,j}\right)}^{2}}{m\times n}},$$

where ${\hat{y}}_{i,j}$ and $y_{i,j}$ are the pixel values of the output and label images, respectively, m×n is the image size of the raw and column images.

## 3. Results

First, the computational time was, on average, 38 h, including training and validation under the set conditions. Consequently, based on the pretrained model, the computational cost was smaller compared to the conventional filtering techniques. 

**Figure 3:**
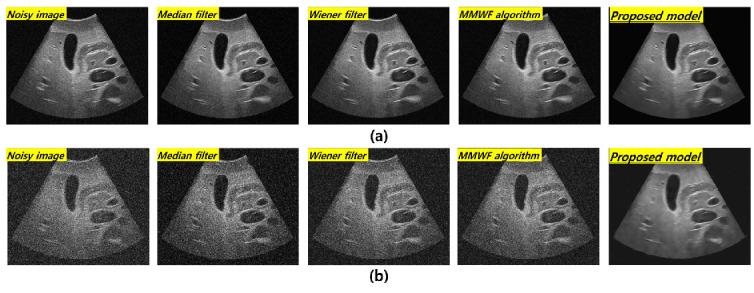
Resulting in images for 3 MHz with (**a**) 0.01 and (**b**) 0.1 standard deviation values of Gaussian and speckle noise distribution corresponding to the noisy image, median filter, Wiener filter, MMWF algorithm, and proposed model, respectively.

**Figure 4:**
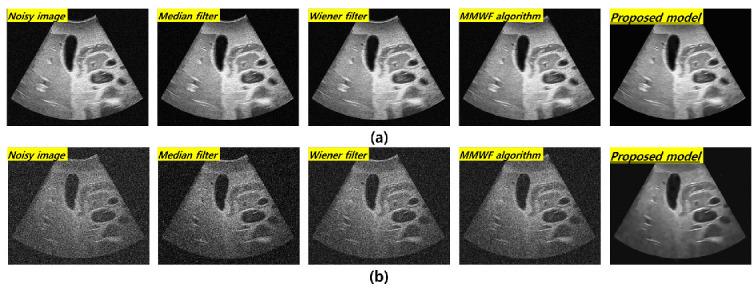
Resulting in images for 5 MHz with (**a**) 0.01 and (**b**) 0.1 standard deviation values of Gaussian and speckle noise distribution corresponding to the noisy image, median filter, Wiener filter, MMWF algorithm, and proposed model, respectively.

Figure 3 shows the resulting images at 3 MHz corresponding to the frequency and standard deviation values of Gaussian and speckle noise in the order of noisy image, median filter, Wiener filter, MMWF algorithm, and the proposed technique with 0.01 and 0.1 standard deviations of the Gaussian and speckle noises, respectively. The visual evaluation indicated an overall improvement in the image quality after applying noise reduction algorithms at 3 MHz. However, the improvement in image quality under the ResNet deep learning algorithm was confirmed at a standard deviation value of 0.1. Figure 4 shows the resulting images corresponding to 0.1 and 0.01 standard deviations of the Gaussian and speckle noise at 5 MHz. The effect of noise reduction can be observed at the 0.1 standard deviation of the fusion noises under the ResNet deep learning model. 

**Figure 5:**
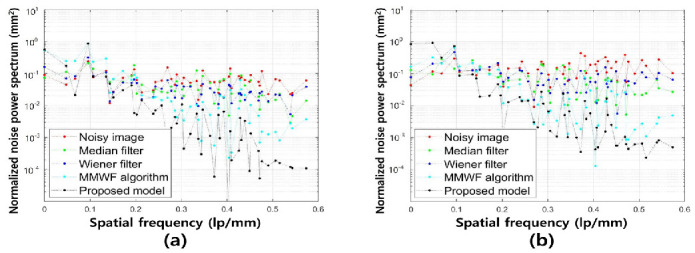
Normalized noise power spectrum results for spatial resolution with (**a**) 0.01 and (**b**) 0.1 of standard deviation values of Gaussian and speckle noise distribution corresponding to noisy, median filter, Wiener filter, MMWF algorithm, and proposed model for 3 MHz.

**Figure 6:**
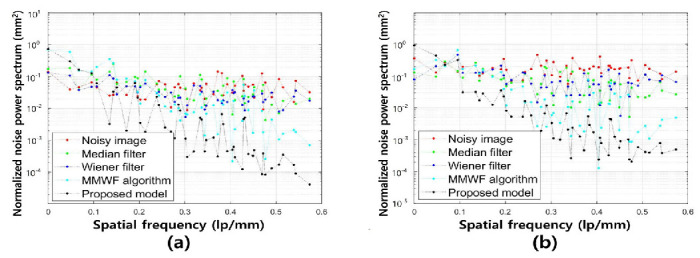
Normalized noise power spectrum results for spatial resolution with (**a**) 0.01 and (**b**) 0.1 of standard deviation values for Gaussian and speckle noise distribution corresponding to noisy, median filter, Wiener filter, MMWF algorithm, and proposed model for 5 MHz.

**Figure 7:**
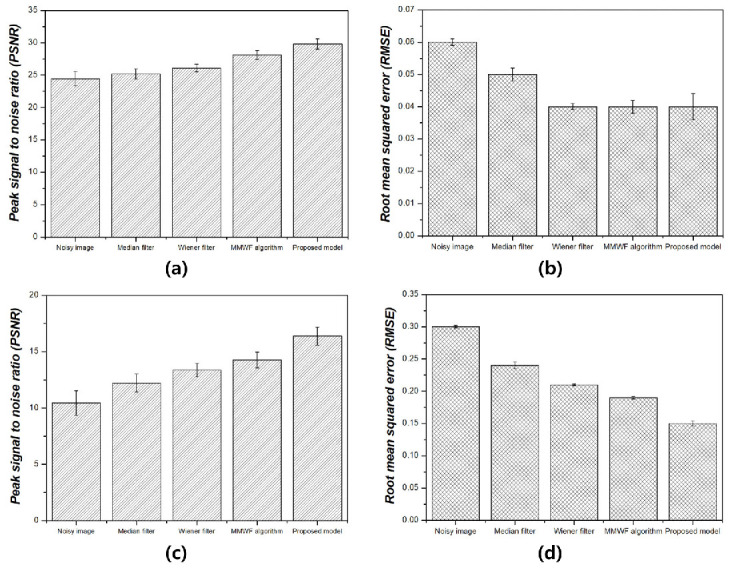
Similarity results between label and output images for 3 MHz for peak signal-to-noise ratio and root mean squared error corresponding to (**a**,**b**) 0.01 and (**c**,**d**) 0.1 of standard deviation values of Gaussian and speckle noise.

**Figure 8:**
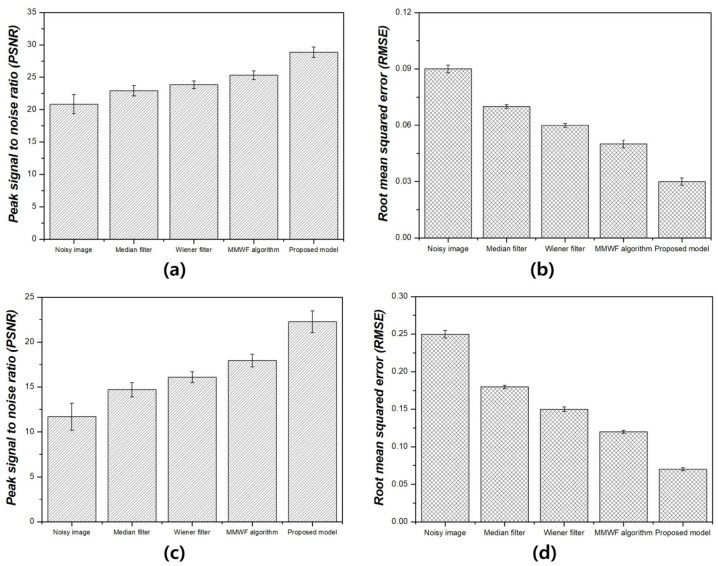
Similarity results between label and output images for 5 MHz for peak signal-to-noise ratio and root mean squared error corresponding to (**a**,**b**) 0.01 and (**c**,**d**) 0.1 standard deviation values of Gaussian and speckle noise.

**Figure 9:**
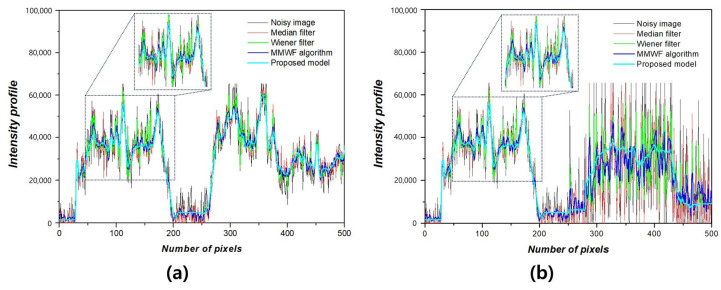
Intensity profile including magnified portion according to (**a**) 0.01 and (**b**) 0.1 standard deviation values of Gaussian and speckle noise at 3 MHz.

**Figure 10:**
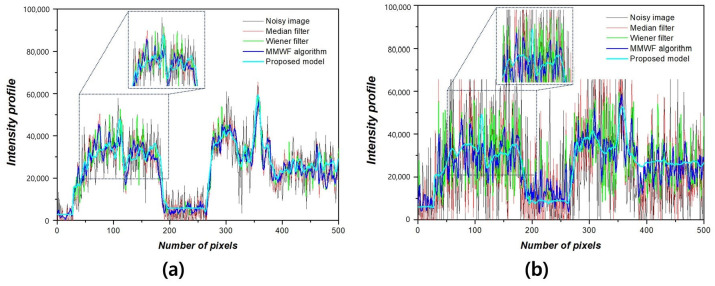
Intensity profile including magnified portion according to (**a**) 0.01 and (**b**) 0.1 standard deviation values of Gaussian and speckle noise at 5 MHz.

Figure 5 shows the NNPS results corresponding to the spatial resolution with 0.01 and 0.1 standard deviation values at 3 MHz. As the spatial resolution increased (0.5 lp/mm), the noise values decreased under all conditions. Overall, the NNPS results indicated that the resulting value was the lowest for the ResNet deep learning model. Figure 6 illustrates the NNPS results corresponding to 0.01 and 0.1 standard deviation values of Gaussian and speckle noise, respectively, at 5 MHz. Based on these results, the distribution of the noise reduction from the noisy image showed the largest decrease for the deep learning algorithm and was in the order of the MMWF algorithm, Wiener filter, and median filter. Figure 7 depicts the similarity results corresponding to the performance evaluation metrics at 3 MHz. Additionally, Figure 7a,b, and Figure 7c,d show the similarity results corresponding to 0.01 and 0.1 standard deviations of Gaussian and speckle noise, respectively. For a standard deviation of 0.01, and for the input, median filter, Wiener filter, MMWF algorithm, and deep learning algorithm, the PSNR values (Figure 7a) were 24.44, 25.20, 26.10, 28.11, and 29.81, respectively, and the RMSE values (Figure 7b) were 0.06, 0.05, 0.04, 0.04 and 0.04, respectively. For a standard deviation of 0.1, and for the input, median filter, Wiener filter, MMWF algorithm, and deep learning algorithm, the PSNR values (Figure 7c) were 10.44, 12.21, 13.36, 14.25, and 16.38, respectively, and the RMSE values (Figure 7d) were 0.30, 0.24, 0.21, 0.19, and 0, respectively. Figure 8 shows the PSNR and RMSE results at 5 MHz with 0.01 and 0.1 standard deviations of Gaussian and speckle noise (Figure 8a,b and Figure 8c,d, respectively). For a standard deviation of 0.01, the PSNR values (Figure 8a) were 20.85, 22.91, 23.84, 25.30, and 28.87, respectively, and the RMSE values (Figure 8b) were 0.09, 0.07, 0.06, 0.05, and 0.03, respectively. For a standard deviation of 0.1, the PSNR values (Figure 8c) were 11.70, 14.71, 16.09, 17.95, and 22.27, respectively, and the RMSE values (Figure 8d) were 0.25, 0.18, 0.15, 0.12, and 0.07, respectively. Figure 9 and Figure 10 show the intensity profile results according to the number of pixels for evaluating the edge information via image processing. As evident from the figures, the proposed model preserves edge information more effectively than other conditions, regardless of the standard deviation values.

## 4. Discussion

The improvement of image quality by reducing the Gaussian and speckle noise in ultrasound imaging is a critical topic. In this study, we developed an alternative denoising method based on a deep learning algorithm and made a comparison with conventional filtering techniques. The ResNet deep learning model was applied to noisy images, including Gaussian and speckle noises, with 0.01 and 0.1 standard deviation values at 3 and 5 MHz, respectively. We acquired deep learning-based denoising images and performed comparisons with the median filter, Wiener filter, and MMWF algorithm using the NNPS, PSNR, and RMSE parameters. 

Based on previous research on noise reduction in ultrasound, the noise reduction task was conducted in various noise environments in the spatial domain using filters such as Gaussian and speckle noise separately [20,21]. Mafi et al. emphasized that evaluating noise reduction is crucial in environments with Gaussian and speckle noise because digital medical imaging, particularly ultrasound imaging, is commonly affected by fusion noise, which includes both speckle and Gaussian noise [10]. Following prior research, we implemented a noise reduction task under fusion noise conditions to achieve accurate results. Furthermore, the frequency characteristic is a key parameter when acquiring ultrasound images of the target organ or tissue. Therefore, we considered the effect of noise reduction under fusion noise, taking into account the degree of standard deviation and both low and high frequencies. 

The denoising task in medical imaging is generally well-established, but research on denoising analysis in ultrasound imaging is lacking compared to other medical imaging modalities such as CT and MRI. This study focuses on reducing fusion noise in ultrasound imaging at two key frequencies, which are critical for acquiring the desired signals from organs. We generated images with combined Gaussian and speckle noises, using standard deviations of 0.01 and 0.1. A novel aspect of our study is the implementation of denoising for fusion noise in ultrasound images acquired at 3 and 5 MHz frequencies. To validate the effectiveness of our deep learning algorithm in noise reduction, we compared and quantitatively analyzed its performance using the ResNet model against conventional algorithms such as the median filter, Wiener filter, and MMWF algorithm. Additionally, we proposed an alternative denoising method based on the ResNet deep learning model.

As compared with conventional deep CNN models such as the VGGNet developed by Simonyan et al., the ResNet model is more accurate and easier to train owing to its deeper architecture [22]. The performance of the ResNet model with 152 deep layers indicated that its structure was simpler compared to the VGGNet. In addition, the ResNet model achieved approximately a 3.8% error rate compared with the ImageNet test set [23]. As evident from Figure 3 and Figure 4, the proposed method is useful for denoising. In other words, the denoising effect was effective at the 0.1 standard deviation value compared to the 0.01 standard deviation value regardless of the 3 and 5 MHz. From Figure 5 and Figure 6, the NNPS results also indicate that the denoising effect is feasible for all set conditions. Figure 7 shows the results of the quantitative similarity analysis between the label image and applied noise reduction algorithms at 3 MHz. The PSNR values under the condition of the deep learning algorithm with 0.01 standard deviation for the Gaussian and speckle noise at 3 MHz were higher than those of the input, median filter, Wiener filter, and MMWF algorithm by approximately 17.9%, 15.4%, 12.4%, and 5.6%, respectively. In addition, the PSNR values under the deep learning algorithm with 0.1 standard deviation at 3 MHz were higher than the input, median filter, Wiener filter, and MMWF algorithm by approximately 36.2%, 25.5%, 18.4%, and 13.0%, respectively. The RMSE results under the 0.01 standard deviation of Gaussian and speckle noise at 3 MHz frequency of the deep learning algorithm were approximately 33.3% and 20.0% lower than those for the input and median filters, respectively, and were nearly identical to the RMSE results of the Wiener filter and MMWF algorithm. In addition, the RMSE results of the deep learning algorithm for the Gaussian and speckle noise with 0.1 standard deviations were lower by approximately 50.0%, 37.5%, 28.6%, and 21.1% compared to those of the input, median filter, Wiener filter, and MMWF algorithm, respectively. Figure 8 shows the results of the similarity analysis for the PSNR and RMSE corresponding to 5 MHz. The PSNR results of the deep learning algorithm under the 0.01 standard deviation for the Gaussian and speckle noise were higher by approximately 27.8%, 20.1%, 17.4%, and 12.4% compared to the input, median filter, Wiener filter, and MMWF algorithm, respectively. In addition, the RMSE results of the deep learning algorithm for the 0.01 standard deviation for the Gaussian and speckle noise were approximately 47.5%, 33.9%, 27.8 5%, and 19.4% lower compared to the input, median filter, Wiener filter, and MMWF algorithm, respectively. The PSNR results of the deep learning algorithm for the 0.1 standard deviation for the Gaussian and speckle noise were higher by approximately 66.7%, 57.1%, 50.0%, and 40.0% compared to the input, median filter, Wiener filter, and MMWF algorithm, respectively. The RMSE results were approximately 72.0%, 61.1%, 53.3%, and 41.7% lower than those of the input, median filter, Wiener filter, and MMWF algorithm, respectively. When comparing the application of the noise distribution, the noise reduction algorithms applied in this study were effective at the 0.1 standard deviation for the Gaussian and speckle noise because the NNPS and RMSE results, which were generally representative of the noise level, were significantly decreased. 

Notably, in this study, the fusion noises with Gaussian and speckle noise with low and high standard deviation values in ultrasound imaging at 3 and 5 MHz can be removed using the ResNet deep learning algorithm. The MMWF algorithm, which is efficient for noise reduction combined with the median and Weiner filters as conventional filtering models, is widely used owing to its high performance while maintaining the edge signal. However, we demonstrated that the noise reduction performance based on the ResNet deep learning model was better compared to that of the MMWF algorithm.

Although the ResNet model has high performance and simple structure, the evaluation of the denoising task using newly developed deep learning models based on the ResNet model needs to be considered. In a recent study by Muhtar et al., the FC-ResNet model (i.e., a modified ResNet-16 model using a convolutional block attention module for improvement of the model generation performance) was developed and demonstrated to be efficient in recognizing multilingual signatures [24]. Thus, comparing the denoising performances of classic ResNet and FC-ResNet models under different noise conditions is also necessary. In the future, we plan to perform the following research steps. In general, higher frequencies in the range of 10–13 MHz are commonly used to examine superficial organs. High-frequency ultrasonic images have a lower transmission depth but higher resolution at low frequencies. Therefore, the noise removal effect at high frequencies should be studied. Notably, verifying the noise removal effect of the proposed deep learning algorithm under the same conditions will be meaningful, as it can facilitate the observation of a difference in noise removal due to frequency changes. Furthermore, we will consider an optimized learning model for clinical application in terms of the denoising task. Still, there are challenges in real-world clinical settings. First, it is difficult to obtain a pair clinical dataset in ultrasound imaging, and we should consider advanced algorithms based on the ResNet model because every patient has different tissues, blood vessels, and disease sites compared to an ultrasound phantom. Additionally, we plan to explore newly advanced deep-learning platforms in medical imaging [25,26,27,28]. In particular, Singh et al. reported that a deep learning algorithm based on vision transformers could diagnose pneumonia on a chest X-ray [25]. In addition, Mohamed et al. suggested a new method for classifying breast cancer using the Nutcracker optimization algorithm based on the chaos game optimization with a cross-vision transformer [27].

## 5. Conclusions

In this study, we analyzed the effectiveness of fusion noise removal using conventional filtering techniques and the ResNet deep learning model. We proposed the ResNet deep learning model as a novel approach. We confirmed the feasibility of the ResNet model in denoising Gaussian and speckle noise with standard deviation values of 0.01 and 0.1 in ultrasound phantom images acquired at 3 and 5 MHz. Compared to conventional filtering techniques, the ResNet model demonstrated superior performance. Based on these results, the previously proposed noise reduction algorithm was effective under all conditions, with the ResNet deep learning model showing the best performance.

## Data Availability

The original contributions presented in the study are included in the article, further inquiries can be directed to the corresponding author/s.

## References

[1] McCollough C.H., Leng S., Yu L., Fletcher J.G. (2015). Dual- and Multi-Energy CT: Principles, Technical Approaches, and Clinical Applications. Radiology.

[2] Zaidi H., Del Guerra A.D. (2011). An outlook on future design of hybrid PET/MRI systems. Med. Phys..

[3] Woods J.C., Wild J.M., Wielpütz M.O., Clancy J.P., Hatabu H., Kauczor H.U., van Beek E.J.R., Altes T.A. (2020). Current state of the art MRI for the longitudinal assessment of cystic fibrosis. J. Magn. Reson. Imaging.

[4] Ballanger B., Jahanshahi M., Broussolle E., Thobois S. (2009). PET functional imaging of deep brain stimulation in movement disorders and psychiatry. J. Cereb. Blood Flow Metab..

[5] Goodman J.W. (1976). Some fundamental properties of speckle. J. Opt. Soc. Am..

[6] Pradeep S., Nirmaladevi P. (2021). A Review on Speckle Noise Reduction Techniques in Ultrasound Medical images based on Spatial Domain, Transform Domain and CNN Methods. IOP Conf. Ser. Master. Sci. Eng..

[7] Liu W., Lin W. (2013). Additive white Gaussian noise level estimation in SVD domain for images. IEEE Trans. Image Process..

[8] Russo F. (2003). A method for estimation and filtering of Gaussian noise in images. IEEE Trans. Instrum. Meas..

[9] Lan Y., Zhang X. (2020). Real-Time Ultrasound Image Despeckling Using Mixed-Attention Mechanism Based Residual UNet. IEEE Access.

[10] Mafi M., Tabarestani S., Cabrerizo M., Barreto A., Adjouadi M. (2018). Denoising of ultrasound images affected by combined speckle and Gaussian noise. IET Image Process..

[11] Singh M., Singh S., Kansal S. Comparative Analysis of Spatial filters for Speckle Reduction in Ultrasound Images. Proceedings of the 2009 WRI World Congress on Computer Science and Information Engineering.

[12] Kumar S., Kumar P., Gupta M., Nagawat A.K. (2010). Performance comparison of median and wiener filter in image de-noising. Int. J. Comput. Appl..

[13] Park J., Song S., Kang S.H., Lee Y. (2024). Performance evaluation of improved median-modified Wiener filter with segmentation method to improve resolution in computed tomographic images. J. Korean Phys. Soc..

[14] Lee S., Lee Y. (2021). Performance evaluation of median-modified Wiener filter algorithm in high-resolution complementary metal–oxide–semiconductor radio-agnetic X-ray imaging system: An experimental study. Nucl. Instrum. Methods Phys. Res. A.

[15] Chang Y., Yan L., Chen M., Fang H., Zhong S. (2020). Two-Stage Convolutional Neural Network for Medical Noise Removal via Image Decomposition. IEEE Trans. Instrum. Meas..

[16] Bengio Y., Simard P., Frasconi P. (1994). Learning log-term dependencies with gradient descent is difficult. IEEE Trans. Neural Netw. Learn. Syst..

[17] He K., Zhang X., Ren S., Sun J. Deep Residual Learning for Image Recognition. Proceedings of the 2016 IEEE Conference on Computer Vision and Pattern Recognition (CVPR).

[18] Ledig C., Theis L., Huszár F., Caballero J., Cunningham A., Acosta A., Aitken A., Tejani A., Totz J., Wang Z. Photo-realistic single image super-resolution using a generative adversarial network. Proceedings of the 2017 IEEE Conference on Computer Vision and Pattern Recognition (CVPR).

[19] Foster F.S., Pavlin C.J., Harasiewicz K.A., Christopher D.A., Turnbull D.H. (2000). Advances in ultrasound biomicroscopy. Ultrasound Med. Biol..

[20] Huang Y.M., Ng M.K., Wen Y.W. (2009). A new total variation method for multiplicative noise removal. SIAM J. Imaging Sci..

[21] Wang S., Huang T.Z., Zhao X.L., Mei J.J., Huang J. (2017). Speckle noise removal in ultrasound images by first- and second-order total variation. Numer. Algorithms.

[22] Simonyan K., Zisserman A. (2014). Very Deep Convolutional networks for Large-Scale Image Recognition. arXiv.

[23] Deng J., Dong W., Socher R., Li L., Li K., Fei-Fei L. ImageNet: A Large-Scale Hierarchical Image Database. Proceedings of the 2009 IEEE Conference on Computer Vision and Pattern Recognition.

[24] Muhtar Y., Muhammat M., Yadikar N., Aysa A., Ubul K. (2023). FC-ResNet: A Multilingual Handwritten Signature Verification Model Using an Improved ResNet with CBAM. Appl. Sci..

[25] Mohamed A.F., Saba A., Hassan M.K., Youssef H.M., Dahou A., Elsheikh A.H., El-Bary A.A., Elaziz M.A., Ibrahim R.A. (2024). Boosted Nutcracker optimizer and Chaos Game Optimization with Cross Vision Transformer for medical image classification. Egypt. Inform. J..

[26] Ukwuoma C.C., Qin Z., Heyat M.B.B., Akhtar F., Smahi A., Jackson J.K., Qadri S.F., Muaad A.Y., Monday H.N., Nneji G.U. (2022). Automated lung-related pneumonia and COVID-19 detection based on novel feature extraction framework and vision transformer approaches using chest X-ray images. Bioengineering.

[27] Singh S., Kumar M., Kumar A., Verma B.K., Abhishek K., Selvarajan S. (2024). Efficient pneumonia detection using Vision Transformers on chest X-rays. Sci. Rep..

[28] Altini N., Brunetti A., Puro E., Taccogna M.G., Saponaro C., Zito F.A., Summa S.D., Bevilacqua V. (2022). NDG-CAM:Nuclei detection in histopathology images with semantic segmentation networks and Grad-CAM. Bioengineering.

